# Preliminary evidence that hydroxyurea is associated with attenuated peripheral sensitization in adults with sickle cell disease

**DOI:** 10.1097/PR9.0000000000000724

**Published:** 2019-03-22

**Authors:** Janelle E. Letzen, Sophie Lanzkron, Kasey Bond, Christopher Patrick Carroll, Jennifer A. Haythornthwaite, Sabrina Nance, Claudia M. Campbell

**Affiliations:** a Department of Psychiatry and Behavioral Sciences, Johns Hopkins University School of Medicine, Baltimore, MD, USA; b Division of Hematology, Johns Hopkins University School of Medicine, Baltimore, MD, USA; c Department of Hematology and Medical Oncology, New York University Langone Health, New York, NY, USA

**Keywords:** Sickle cell disease, Hydroxyurea, Quantitative sensory testing, VOC pain, Chronic pain

## Abstract

**Introduction:**

Hydroxyurea (HU) is a drug that targets the underlying pathophysiology of sickle cell disease (SCD); however, it continues to be an underutilized treatment for adults. Previous research suggests that HU treatment can result in fewer hospital contacts for acute vaso-occlusive pain crises (VOC). Hydroxyurea's impact on non-VOC pain, however, is not well established.

**Objectives:**

This study examined whether HU moderated patterns of static and dynamic pain processing and clinical pain in SCD individuals.

**Methods:**

Fifty-eight patients with SCD (N taking HU = 17) underwent quantitative sensory testing (QST) and completed twice daily symptom diaries for 12 weeks. Quantitative sensory testing established thermal threshold and tolerance, mechanical thresholds, and thermal and mechanical temporal summation of pain.

**Results:**

Groups did not differ in age, sex, or opioid use. After controlling for morphine use, QST results showed that participants taking HU had higher heat and mechanical pain thresholds (static QST measures) but not thermal and mechanical temporal summation (dynamic QST measures). Participants taking HU also reported lower VOC pain compared with SCD participants not taking HU; however, HU did not moderate non-VOC clinical pain ratings.

**Conclusion:**

Findings cautiously suggest that HU acts on pain hypersensitivity and VOC pain, rather than inhibiting pain facilitation and non-VOC pain. These differences may reflect HU's influence on peripheral rather than central sensitization. Future research is warranted to replicate these findings in a larger sample and determine whether early HU administration can prevent peripheral sensitization in SCD individuals.

## 1. Introduction

Sickle cell disease (SCD) is a collection of congenital hemoglobinopathies that causes erythrocyte malformations. These “sickled” cells promote hemolysis, vaso-occlusion, and tissue hypoxia,^11^ leading to a myriad of health complications. Episodes of severe, acute pain, classically attributed to vaso-occlusive crises (VOC), are a hallmark of the disease. Up to 65% of adult patients also experience chronic pain.^30^ Whereas VOC pain is acute, episodic, and associated with ischemic events, the etiology of noncrisis chronic pain (ie, non-VOC pain) is complex and poorly understood.^16^

Central sensitization (CS), or plasticity of neurons in response to inflammation or injury,^34^ is one possible cause of non-VOC pain.^3,13^ Contemporary theory suggests peripheral neuron hyperexcitability—likely triggered by VOCs, organ complications, and opioid use—might have compounding effects on central pain modulation systems,^32^ contributing to the amplification and facilitation of nociception.^22^ In patients with SCD, previous work suggests that CS is associated with lower fetal hemoglobin levels,^17^ which is a modulator of SCD severity.^4^ Treatments targeting fetal hemoglobin might reduce CS and non-VOC pain^17^; however, no study has previously examined such effects.

Hydroxyurea (HU), an FDA-approved treatment, decreases the proportion of sickled cells by increasing fetal hemoglobin,^10^ which reduces hemoglobin polymerization and inhibits noxious enzymes.^2^ Positive outcomes associated with HU include reductions in early mortality,^29^ acute VOCs,^15^ and daily symptom interference.^31^ Because few studies have examined HU's impact on non-VOC pain,^9^ this study tested the hypotheses that (1) non-VOC pain would be less severe in SCD adults taking HU (HU+) compared with those not taking HU (HU−), and (2) CS, captured by quantitative sensory testing (QST),^5^ would be reduced among HU+ individuals.

## 2. Methods

This secondary analysis uses data from a case–control protocol examining pain processing and daily function in SCD adults.^12–14,24^ Previously reported sample sizes vary based on data collection stage and aim. This study uniquely focuses on HU's association with evoked and clinical pain to address the knowledge gap about HU and non-VOC pain.^9^ Hydroxyurea is indicated for individuals with hemoglobin (Hb) SS and Hb Beta^0^ thalassemia, so this study only includes data from participants with these genotypes (n = 58). The Institutional Review Board for Johns Hopkins University approved all study-related procedures, and all participants provided written consent.

### 2.1. Data collection procedures

Inclusion and exclusion criteria are listed in our previous work.^12–14,24^ Phone-screened eligible individuals attended an in-person visit on a day of typical pain (but <5/10 intensity) with no VOCs in the previous 3 weeks. Consented participants completed questionnaires, QST, and daily electronic diaries for 12 weeks.

### 2.2. Measures

#### 2.2.1. Quantitative sensory testing

Detailed QST procedures were previously described (see Refs. 12 and 13). Briefly, we assessed pain threshold and tolerance (ie, static QST measures) as well as temporal summation of pain (ie, dynamic QST measures). Heat pain threshold and tolerance stimuli were delivered using a Peltier element-based stimulator (Medoc, Israel; Pathway, Advanced Thermal Stimulator thermode) applied on the ventral forearm with an ascending method of limits paradigm that had a 0.5°/sec rise rate. Mechanical pain threshold stimuli were delivered twice each using an algometer (SBMedic) with a 1-cm^2^ hard rubber probe applied bilaterally on the trapezius muscle, interphalangeal joint of the thumb, the proximal third of the forearm, and the middle of the quadriceps insertion point.

Thermal temporal summation was assessed through the PATHWAY CHEPS (Contact Heat-Evoked Potential Stimulator) thermode using 10 heat pulses spaced by 2.5 seconds with a rise rate of 70°/sec. Mechanical temporal summation was assessed through hand-crafted punctate probes according to the German Research Network protocol. Participants indicated peak pain over 10 stimuli with varying forces between 128 mN and 256 mN delivered 1 second apart.

#### 2.2.2. Daily electronic symptom monitoring

Participants completed daily diaries for 12 weeks after the visit, providing daily average pain intensity ratings on a 0 to 100 scale. Pain ratings were categorized as “VOC” or “non-VOC” based on separately averaged ratings for crisis and noncrisis days, respectively. Data from individuals with ≥25% completed days were included.

### 2.3. Data analyses

The sample was dichotomized based on reported HU use. Skewed and kurtotic variables were log-transformed. Quantitative sensory testing measures and clinical pain were compared using analysis of covariance to control for opioid use. Because this study represents a preliminary analysis of a novel topic, we did not correct for multiple comparisons to minimize the likelihood of type II error^6,18,27^; results should be interpreted cautiously.

## 3. Results

### 3.1. Participants

Of the 58 participants, 17 endorsed regular HU use (SS genotype = 16). Table 1 reports demographic characteristics. All individuals were African American/black, and one individual identified as Hispanic (HU− group). Chi-squared tests showed no group differences in sex (*P* = 0.984), ethnicity (*P* = 0.519), and education (*P* = 0.241). There were no differences in age (*P* = 0.14) or daily morphine equivalents (*P* = 0.431). HU+ participants had significantly lower white blood cell counts than HU− participants (t_56_ = −3.1, *P* = 0.004), demonstrating HU's therapeutic efficacy.

**Table 1:**
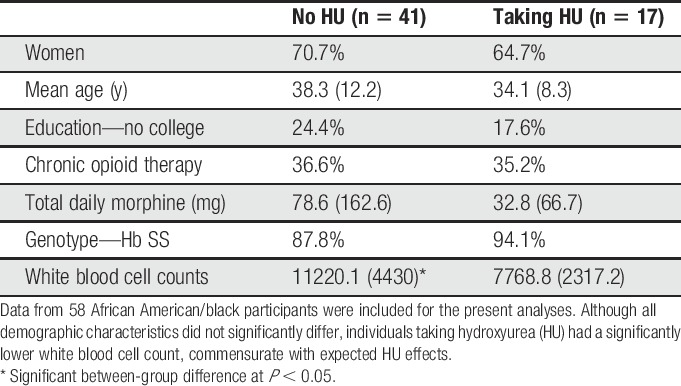
Participant demographic information.

### 3.2. Quantitative sensory testing

**Figure 1.**
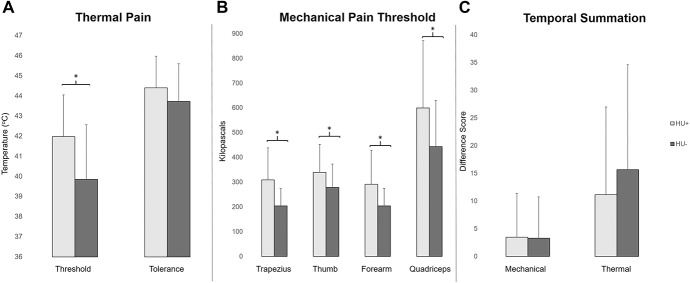
Quantitative sensory testing (QST). Sickle cell disease participants who were and were not taking HU (HU+ and HU−, respectively) underwent static and dynamic QST using thermal and mechanical stimulation. (A) Hydroxyurea+ participants (light gray bars) had significantly higher thermal pain threshold but not tolerance. (B) Further, HU+ individuals evidenced significantly higher mechanical pain thresholds across all four testing sites. (C) However, dynamic QST measures did not differ between groups, suggesting equivocal levels of pain facilitation for HU+ and HU− individuals (error bars represent SDs; *P < 0.05). HU, hydroxyurea.

Figure 1 shows results from analyses of covariance comparing groups on measures of static (Fig. 1A, B) and dynamic (Fig. 1C) pain processing. Total daily morphine equivalents were positively associated with mechanical pain threshold in the thumb (*P* = 0.03), quadriceps (*P* = 0.02), and forearm (*P* = 0.08, trend) but not heat pain threshold/tolerance (*P* = 0.98, 0.63, respectively), trapezius pain threshold (*P* = 0.23), or temporal summation (*P*s > 0.12). On static QST measures, HU+ individuals demonstrated significantly higher thermal pain threshold (F_1,52_ = 9.2, *P* = 0.004, 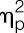 = 0.15) but not tolerance (F_1,51_ = 1.3, *P* = 0.25, 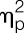 = 0.03) compared with HU− participants. HU+ individuals also had significantly higher mechanical pain thresholds in the trapezius (F_1,52_ = 14.3, *P* < 0.001, 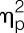 = 0.22), forearm (F_1,52_ = 9.7, *P* = 0.003, 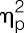 = 0.16), thumb (F_1,51_ = 7.0, *P* = 0.01, 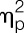 = 0.12), and quadriceps (F_1,52_ = 9.1, *P* = 0.004, 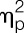 = 0.15). However, there were no group differences in thermal or mechanical temporal summation (thermal: F_1,52_ = 0.75, *P* = 0.4, 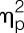 = 0.02; mechanical: F_1,48_ = 0.03, *P* = 0.86, 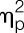 = 0.0).

### 3.3. Clinical pain ratings

**Figure 2.**
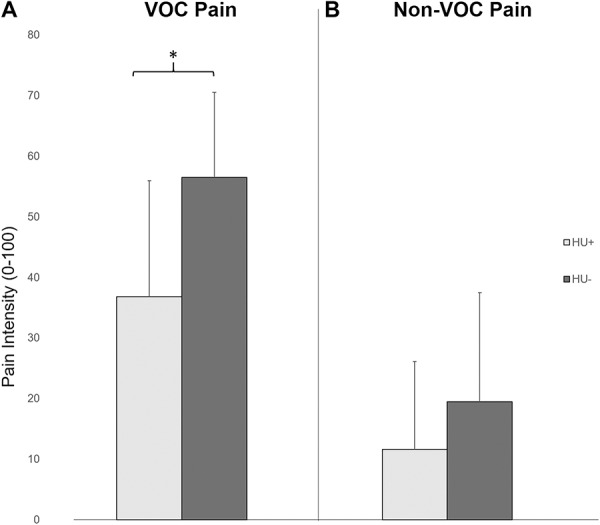
Clinical pain ratings based on responses twice-daily electronic symptom diaries over a 12‐week period were examined using independent‐sample t‐tests. (A) Individuals reporting hydroxyurea use (HU+) had significantly lower VOC pain. (B) However, non‐VOC pain did not significantly differ between groups (error bars represent SDs; *P < 0.05). HU, hydroxyurea; VOC, vaso‐occlusive crises.

Forty-seven percent of HU+ and 63% of HU− participants reported non-VOC pain (Fisher's *Z* = 1.7, *P* = 0.1). Twenty-four percent of HU+ and 46% of HU− participants reported VOC pain (Fisher's *Z* = 1.6, *P* = 0.2). Total daily morphine equivalents were positively associated with non-VOC (*P* = 0.003) and VOC pain (*P* = 0.01). After controlling for this factor, non-VOC pain did not significantly differ between HU+ and HU− individuals (F_1,49_ = 1.4, *P* = 0.25, 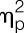 = 0.03), but HU+ participants reported significantly lower VOC pain ratings (F_1,31_ = 10.4, *P* = 0.003, 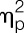 = 0.25). Table 2 provides QST and clinical pain descriptive statistics, and Figure 2 depicts differences in diary pain ratings averaged over a 12-week period.

**Table 2:**
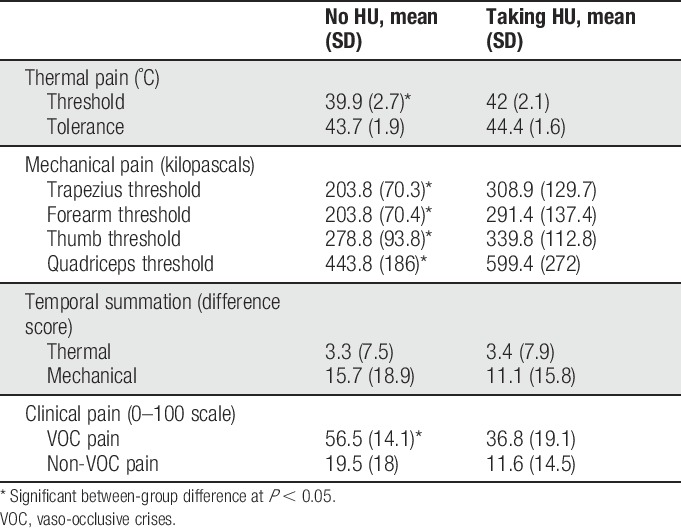
Descriptive information for quantitative sensory testing and clinical pain measures comparing individuals taking and not taking hydroxyurea (HU).

## 4. Discussion

Findings from this study did not support our hypotheses. HU+ participants did not report significantly lower non-VOC pain on daily diaries, nor did they show evidence of reduced pain facilitation on dynamic QST measures. Instead, HU+ participants reported significantly lower VOC pain and had lower pain thresholds on static QST measures (ie, thermal pain and mechanical pain across the 4 testing sites) compared with HU− individuals. This effect remained after controlling for total daily morphine equivalents, suggesting that opioid use did not confound the present results.

Previous work supports our finding of reduced VOC pain ratings in SCD individuals treated with HU.^2,7,10,15,21,33^ Hydroxyurea's ability to increase levels of fetal hemoglobin,^15,19^ change erythrocyte properties,^8,26^ and decrease cell adhesion^1^ results in reduction of inflammatory mediators in the periphery.^23^ It is possible, then, that our finding of lower static QST ratings among HU+ participants reflects treatment-induced changes in peripheral pain mechanisms.

Alternatively, we did not observe differences in non-VOC pain ratings, nor dynamic QST measures. Non-VOC pain in SCD has been described as a potential interaction of peripheral and CS mechanisms.^20^ Furthermore, the selected dynamic QST measures are used to probe sensitization of the central nervous system.^28^ Combined, these findings suggest that CS is not directly influenced through HU treatment. Future, large-scale studies yielding normative QST values for individuals with SCD will help track changes in pain processing over the course of HU treatment.

## 5. Limitations

Our study had limitations for future work to expand upon. First, this study is a small, secondary analysis with cross-sectional reporting of HU use. Sickle cell disease is a rare disorder in the United States, which limits sample sizes. Second, we did not collect information about HU treatment duration or dosing over time. It is possible that long-term or early-initiated treatment impacts CS progression. Given the study's cross-sectional nature, we cannot speak to whether early HU initiation might prevent the development of non-VOC pain. Third, we did not collect information about adherence to HU, which might confound outcomes.^7^ Finally, future research is encouraged to examine a larger cohort of individuals taking HU to address this study's limitation of unequal sample sizes. Although these limitations support cautious interpretation of these results, findings suggest that HU more robustly impacts VOC than non-VOC pain mechanisms.

## 6. Conclusion

Non-VOC pain remains understudied^30^ and, critically, powerfully impacts quality of life in individuals with SCD.^25^ The present results suggest that HU is not associated with lower non-VOC pain but is robustly related to better VOC outcomes. Future research is encouraged to establish alternative treatments acting on central pain mechanisms in SCD to address non-VOC pain.

## Disclosures

The authors have no conflict of interest to declare.
